# Reduced Nectin-4 expression in lymph node metastases of penile squamous cell carcinoma: a paired primary–metastatic analysis

**DOI:** 10.3389/fonc.2026.1912533

**Published:** 2026-09-22

**Authors:** Tianhao Ma, Kun Wang, Feiran Chen, Hongcheng Zhao, Chunsen Yang, Buwei Wu, Xin Yao

**Affiliations:** Key Laboratory of Cancer Prevention and Therapy, Department of Urologic Oncology, National Clinical Research Center for Cancer, Tianjin’s Clinical Research Center for Cancer, Tianjin Medical University Cancer Institute and Hospital, Tianjin, China

**Keywords:** immunohistochemistry, lymph node metastasis, Nectin-4, penile squamous cell carcinoma, prognosis

## Abstract

**Objective:**

To evaluate Nectin-4 expression in primary and matched metastatic penile squamous cell carcinoma (PSCC) lesions and to assess its associations with clinicopathological features and survival outcomes.

**Methods:**

We analyzed 123 patients with pathologically confirmed PSCC treated at our hospital between 2018 and 2024. Nectin-4 expression was assessed by immunohistochemistry using the H-score (0-300) in primary tumors and 30 matched lymph node metastases, and associations with pathological features, paired primary-metastatic differences, and survival outcomes were analyzed.

**Results:**

Nectin-4 was widely expressed in PSCC. Higher Nectin-4 expression was observed in less aggressive tumors. Expression was higher in T1 than in T2 stage (95% CI 19.10-79.28; P < 0.01) and decreased from G1 to G3, with significant differences between G1 and G2 (95% CI 0.39-63.83; P = 0.047) and between G1 and G3 (95% CI 24.41-170.43; P = 0.009). Expression in primary tumors was not significantly associated with nodal status. In paired samples, lymph node metastases showed lower H-scores than their matched primary tumors (95% CI 3.52-72.82; P = 0.032). Low Nectin-4 expression was associated with shorter overall survival in Kaplan-Meier and univariable Cox analyses, but it was not independently associated with survival after multivariable adjustment.

**Conclusions:**

Nectin-4 expression in PSCC is higher in early, better-differentiated tumors and lower in lymph node metastases. Low Nectin-4 expression was associated with poorer overall survival, although it was not an independent prognostic factor. These findings suggest that Nectin-4 may have prognostic relevance and potential value as a therapeutic target in PSCC.

## 1 Introduction

Penile squamous cell carcinoma (PSCC) is a relatively rare but highly aggressive malignancy, with a disproportionately high incidence in certain developing regions (1, 2). Despite surgical advances and improved understanding of HPV-related and HPV-independent tumorigenesis (3), the prognosis for patients with regional lymph node metastasis or advanced disease remains poor (4). While regional lymph node involvement is the primary prognostic indicator (5), previous research has failed to establish reliable biomarkers for predicting tumor progression, metastatic potential, or therapeutic response (6). Therefore, the identification of molecular markers that improve risk stratification and have therapeutic implications is crucial.

Nectin-4 (PVRL4), a member of the immunoglobulin superfamily, is a cell adhesion molecule crucial for mediating cell-cell adhesion, maintaining epithelial integrity, and supporting tumor-stroma interactions (7). Accumulating evidence has implicated Nectin-4 in the pathobiology of multiple epithelial malignancies, with overexpression reported in breast, ovarian, lung, and urothelial carcinomas (8). Its expression is closely associated with tumor growth, immune regulation, and metastatic progression, positioning it as a promising cell-surface therapeutic target (9). The Nectin-4-targeting antibody–drug conjugate (ADC) enfortumab vedotin has demonstrated substantial clinical benefit in metastatic urothelial carcinoma (10). Ongoing phase II trials are evaluating enfortumab vedotin as monotherapy in locally advanced or metastatic PSCC (NCT06104618) and in combination with avelumab as first-line treatment for advanced or metastatic penile carcinoma (NCT07110038), further supporting the translational relevance of Nectin-4-targeted therapy in this disease (11, 12). Despite its established significance in various solid tumors, the expression patterns and clinical implications of Nectin-4 in PSCC remain poorly understood.

Recent studies have documented Nectin-4 expression in PSCC, although the findings are inconsistent, with substantial variability in reported positivity rates and scoring criteria across studies (13). Systematic evaluation of its biological and clinical significance is scarce. This limitation is particularly evident in the context of metastasis, as most available studies have focused on primary tumors, while a comprehensive evaluation of Nectin-4 expression in lymph node metastases remains lacking (14, 15). It remains unclear whether Nectin-4 expression changes during PSCC progression and metastasis and how such changes may affect its value as a prognostic biomarker or therapeutic target. Emerging evidence from urothelial carcinoma indicates that the expression of antibody–drug conjugate targets is influenced by tumor differentiation states and lineage-associated transcriptional programs (16–18). Whether a comparable process occurs in PSCC remains unknown. Given that metastatic progression may be accompanied by remodeling of epithelial differentiation and cell-surface molecules, defining Nectin-4 expression in matched primary and metastatic PSCC lesions may help determine whether it remains a meaningful therapeutic target in advanced disease.

Therefore, we examined Nectin-4 expression in primary PSCC lesions and matched lymph node metastases to characterize how its expression changes during disease progression and metastasis. By correlating semi-quantitative H-scores with key clinicopathological parameters—including T stage, histological grade, and nodal status—we aimed to assess the clinicopathological and prognostic relevance of Nectin-4 and its potential implications for Nectin-4-targeted therapy in PSCC.

## 2 Materials and methods

### 2.1 Patient cohort

This single-center retrospective study initially screened 189 patients with pathologically confirmed penile squamous cell carcinoma (PSCC) who underwent surgical treatment at the Department of Urologic Oncology, Tianjin Medical University Cancer Institute and Hospital between March 2018 and December 2024. Patients were excluded because of unavailable primary-tumor FFPE blocks (n = 50), receipt of neoadjuvant therapy (n = 7), or inadequate specimen preservation or quality for analysis (n = 9), resulting in 123 patients in the primary-tumor cohort. Among the 35 patients with pathologically confirmed regional lymph node metastasis (pN1–3), metastatic lymph node FFPE blocks were available and adequate for immunohistochemical evaluation in 30 patients. These patients constituted the paired primary–metastatic cohort. The remaining five patients were excluded only from the paired analysis because metastatic lymph node FFPE tissue were unavailable (**Supplementary Figure 1**). Tumor stage was assigned according to the 8th edition of the American Joint Committee on Cancer (AJCC) TNM staging system. pN0 was defined as pathologically confirmed absence of regional lymph node metastasis, whereas pN1–3 indicated pathologically confirmed regional lymph node metastasis. Patients without pathological assessment of regional lymph nodes were classified as pNx. Patients with pNx disease were retained in analyses that did not require pathological nodal status but were excluded from analyses in which pathological nodal status was evaluated.

### 2.2 Immunohistochemistry

Nectin-4 expression was assessed by immunohistochemistry (IHC) in 123 primary tumor specimens and 30 matched FFPE specimens from lymph node metastases. Immunostaining was conducted using a monoclonal antibody against Nectin-4 (Abcam, ab192033) at a dilution of 1:100. Staining was performed on the VENTANA BenchMark ULTRA platform according to standardized protocols. Nectin-4 immunohistochemistry was evaluated on one representative whole-section slide from an FFPE block for each primary or metastatic lesion. Only membranous staining in tumor cells was included in the assessment. Each slide was initially examined at low magnification to identify the entire evaluable tumor area. Nectin-4 staining intensity in tumor cells was classified as negative (0), weak (1+), moderate (2+), or strong (3+), as illustrated in **Supplementary Figure 2**. The H-score was calculated as follows: H-score = [1 × percentage of weakly stained cells] + [2 × percentage of moderately stained cells] + [3 × percentage of strongly stained cells], resulting in a score ranging from 0 to 300. Two experienced pathologists who were blinded to the clinicopathological and survival data independently evaluated all slides. Interobserver agreement for H-scores was evaluated using the intraclass correlation coefficient (ICC) with 95% confidence intervals. The mean of the two independently assigned H-scores was used for analysis. Cases with marked discrepancies were jointly reviewed by the two pathologists to reach a consensus.

### 2.3 Statistical analysis

Overall survival (OS) and disease-free survival (DFS) were the main survival endpoints of this study. OS was calculated from the date of penectomy to the date of death from any cause or the last follow-up. DFS was defined as the interval from the date of penectomy to the first documented recurrence (local, regional, or distant) or death from penile cancer. Patients without an event were censored at the date of the last follow-up. DFS analysis was restricted to patients who achieved no evidence of disease (NED) after initial curative-intent treatment.

Statistical analyses were performed using SPSS software (version 27.0; IBM Corp., Armonk, NY, USA). Continuous variables are presented as the mean ± standard deviation for normally distributed variables or median with interquartile range for nonnormally distributed variables. Group comparisons were conducted using parametric (independent-samples t-test, one-way ANOVA, paired-samples t-test) or non-parametric tests (Mann–Whitney U test, Kruskal–Wallis test) based on data distribution. For comparisons involving more than two groups, Bonferroni-adjusted *post hoc* pairwise comparisons were performed, and adjusted P values are reported. The correlation between primary and metastatic H-scores was assessed using Pearson’s correlation coefficient. The Kaplan–Meier method was used to estimate survival curves, while the log-rank test was applied to assess differences between groups. Univariate Cox proportional hazards regression analysis was performed to evaluate the association between clinicopathological variables and OS. Variables with P < 0.05 in univariate analysis were considered for inclusion in the multivariate Cox regression model. Given the limited number of OS events, the model was not further expanded with additional covariates to reduce the risk of overfitting; therefore, the final model included only pathological nodal status and continuous Nectin-4 H-score. All statistical tests were two-sided, and P < 0.05 was considered statistically significant.

## 3 Results

### 3.1 Patient characteristics

A total of 123 patients with PSCC were included in this study (**Table 1**). The mean age was 59.89 years, and the median follow-up was 32 months. T1–T2 disease accounted for 83.8% of cases (43 and 60 cases, respectively), whereas T3–T4 disease accounted for 12.2%. Regarding lymph node status, 53.7% of patients were classified as N0. Histological grade was predominantly G1–G2, accounting for 80.5% of cases. Nectin-4 expression was high in a substantial proportion of tumors. Specifically, 67.5% of cases showed positive staining in more than 75% of tumor cells, and H-scores most frequently fell within the 100–200 range (43.9%). The two pathologists showed excellent agreement in Nectin-4 H-score assessment (ICC = 0.92, 95% CI, 0.89–0.94). The clinicopathological and expression characteristics of the 30 paired primary and metastatic cases are summarized in **Supplementary Table 1**.

**Table 1:** Clinicopathological characteristics of the patient cohort (N = 123).

Characteristic	N	%
Age (years), mean ± SD	59.89 ± 12.13	
Median follow-up, months median (IQR)	32 (13-63)	
Smoking	72	
Underlying disease		
Hypertension	41	
Diabetes	23	
Coronary artery disease	12	
Surgery procedure		
Partial penectomy	107	87.0
Total penectomy	13	10.6
Wide excision	3	2.4
Regional nodal management		
No pathological nodal assessment	22	17.9
ILND	86	69.9
ILND + PLND	15	12.2
Adjuvant treatment		
Chemotherapy	33	26.8
None	90	73.2
Surgical margin status		
Negative	123	100.0
Positive	0	0
Primary tumor		
T1	43	35.0
T2	60	48.8
T3	13	10.6
T4	2	1.6
Tx	5	4.0
pN stage		
N0	66	53.7
N1	11	8.9
N2	16	13.0
N3	8	6.5
Nx	22	17.9
Histological grade		
G1	38	30.9
G2	61	49.6
G3	5	4.1
Gx	19	15.4
Proportion of positive tumor cells		
0-25%	9	7.3
25-50%	16	13.0
50-75%	15	12.2
75-100%	83	67.5
H-score		
0-100	39	31.7
100-200	54	43.9
200-300	30	24.4

### 3.2 Association between Nectin-4 expression and clinicopathological characteristics

In primary tumors, Nectin-4 expression was associated with T stage and histological grade, but not with nodal status. Expression levels were higher in early-stage tumors (**Figure 1A**). The difference between the T1 and T2 groups was statistically significant (95% CI 19.10-79.28; P < 0.01). H-scores were also lower in T3–4 tumors, although the difference did not reach statistical significance.

**Figure 1:**
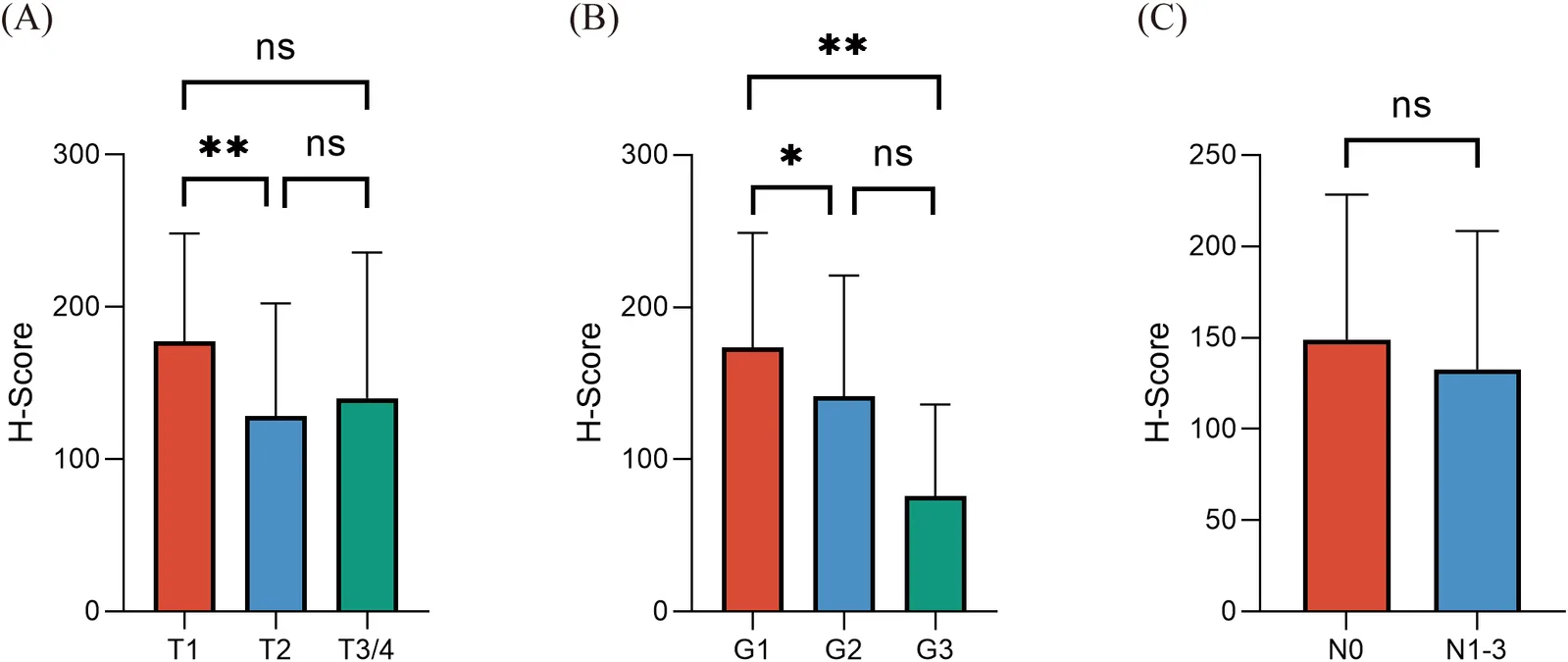
Nectin-4 expression in PSCC according to clinicopathological features. Nectin-4 H-scores in different groups: **(A)** T stage (T1, T2, and T3/4), **(B)** histological grade (G1, G2, and G3), and **(C)** lymph node status (N0 and N1–3). Bars represent mean ± SD. ns, not significant; *P < 0.05; **P < 0.01.

Nectin-4 expression decreased with increasing histological grade. H-scores were significantly higher in G1 tumors than in G2 (95% CI 0.39-63.83; P = 0.047) and G3 tumors (95% CI 24.41-170.43; P = 0.009), whereas no significant difference was observed between the G2 and G3 groups (**Figure 1B**).

Nectin-4 expression in primary tumors was not significantly associated with lymph node status. H-score distributions largely overlapped between patients with and without nodal metastasis (**Figure 1C**).

### 3.3 Differences in Nectin-4 expression between primary tumors and lymph node metastases

Among the 30 patients with paired specimens from primary tumors and lymph node metastases, Nectin-4 expression was significantly lower in metastatic lesions (**Figure 2A**). The mean H-score in metastatic lesions was 38.17 points lower than that in the corresponding primary tumors (95% CI 3.52-72.82; P = 0.032). In 23 paired cases, metastatic lesions showed lower H-scores than matched primary tumors, whereas the remaining 7 cases (23.3%) showed higher H-scores (**Figure 2A**). Primary and metastatic H-scores showed a weak positive correlation, although it was not statistically significant (r = 0.275, P = 0.141). The proportion of cases with an H-score of 0–100 increased from 36.7% (11/30) in primary tumors to 56.7% (17/30) in metastatic lesions, whereas the proportion of cases with an H-score of 200–300 decreased from 20.0% (6/30) to 3.3% (1/30). Despite the significant reduction in Nectin-4 expression in metastatic lesions relative to primary tumors, expression was not completely lost. In 80.0% of metastatic cases, more than 25% of tumor cells were Nectin-4-positive, and 43.3% of metastatic lesions showed positive staining in more than 75% of tumor cells (**Figure 2B**).

**Figure 2:**
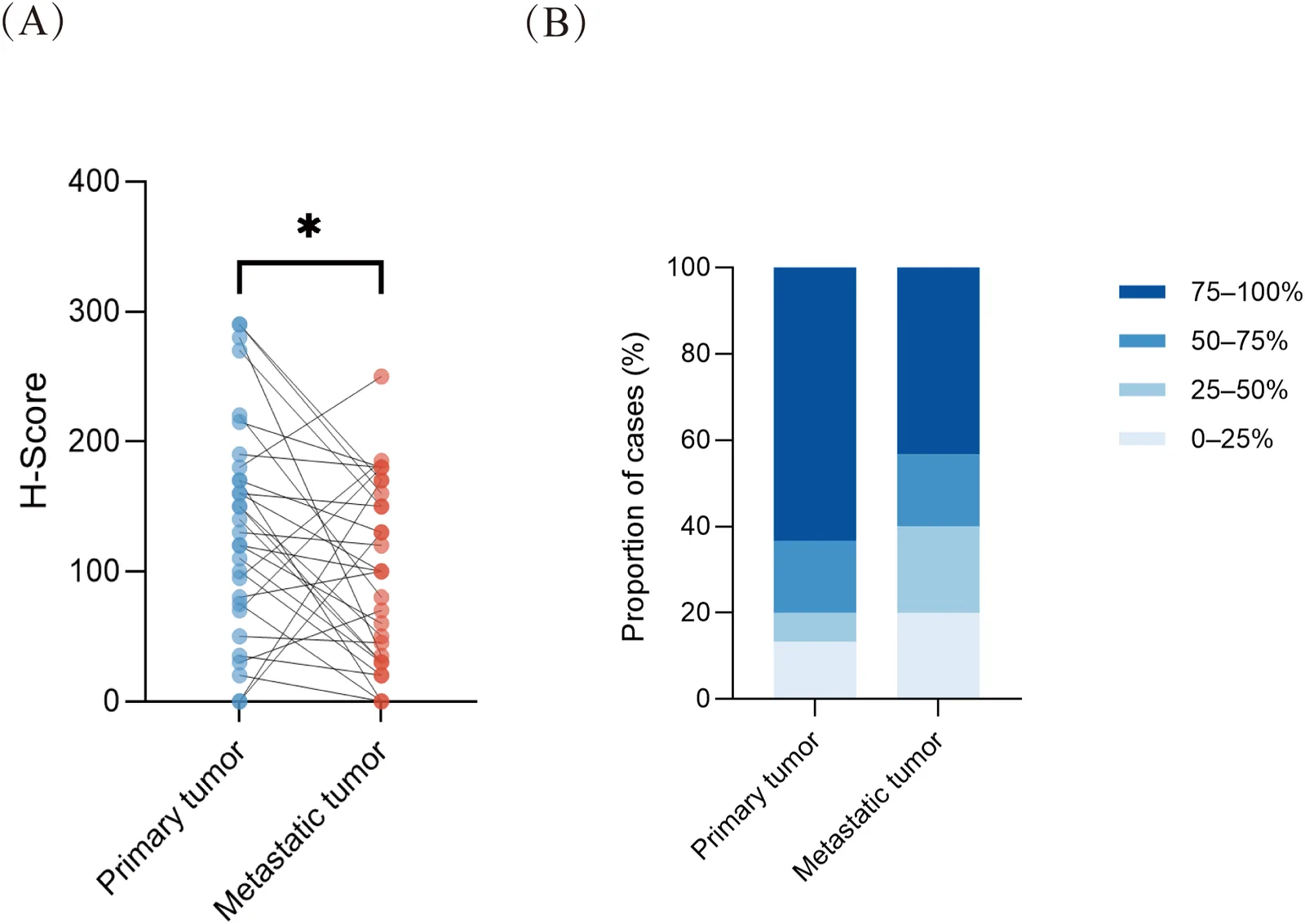
Nectin-4 expression in matched primary tumors and lymph node metastases of penile squamous cell carcinoma. **(A)** Paired comparison of Nectin-4 H-scores between primary tumors and corresponding lymph node metastases. **(B)** 100% stacked bar plot showing the distribution of cases according to the proportion of Nectin-4-positive tumor cells (0–25%, >25–50%, >50–75%, and >75–100%) in primary tumors and matched lymph node metastases (n = 30 each). *P < 0.05.

### 3.4 Prognostic analysis

During follow-up, 22 deaths and 29 DFS events were observed. All 123 patients achieved no evidence of disease after initial treatment and were therefore eligible for the DFS analysis. The numbers of events in each Nectin-4 group and the analytic sample sizes are summarized in **Supplementary Table 2**. Kaplan–Meier analysis showed a significant difference in overall survival (OS) between the high- and low-expression groups defined by the median H-score of 150 (**Figure 3**). Low Nectin-4 expression was significantly associated with shorter OS (P = 0.0173). DFS was also numerically shorter in the low-expression group, but the difference was not statistically significant (P = 0.3531). The results of Cox regression analyses for OS are shown in **Table 2**. Univariate Cox regression analysis showed that positive nodal status was significantly associated with poorer OS (HR = 12.631, 95% CI 2.680–59.536; P = 0.001). When H-score was analyzed as a continuous variable, higher Nectin-4 expression was associated with a lower risk of death in univariable Cox regression analysis (HR = 0.951, 95% CI 0.905–1.000; P = 0.048).T stage and histological grade showed borderline associations with OS, whereas age was not significantly associated with OS. After excluding 22 patients with pNx disease, 101 patients were included in the multivariate Cox regression analysis. Positive nodal status remained independently associated with poorer OS (HR = 10.627, 95% CI 2.197-51.329; P = 0.003). After adjustment for pathological nodal status, Nectin-4 H-score was not independently associated with OS (HR = 0.995, 95% CI 0.989–1.001; P = 0.108).Accordingly, the present data support prognostic relevance but do not establish Nectin-4 as an independent prognostic biomarker.

**Figure 3:**
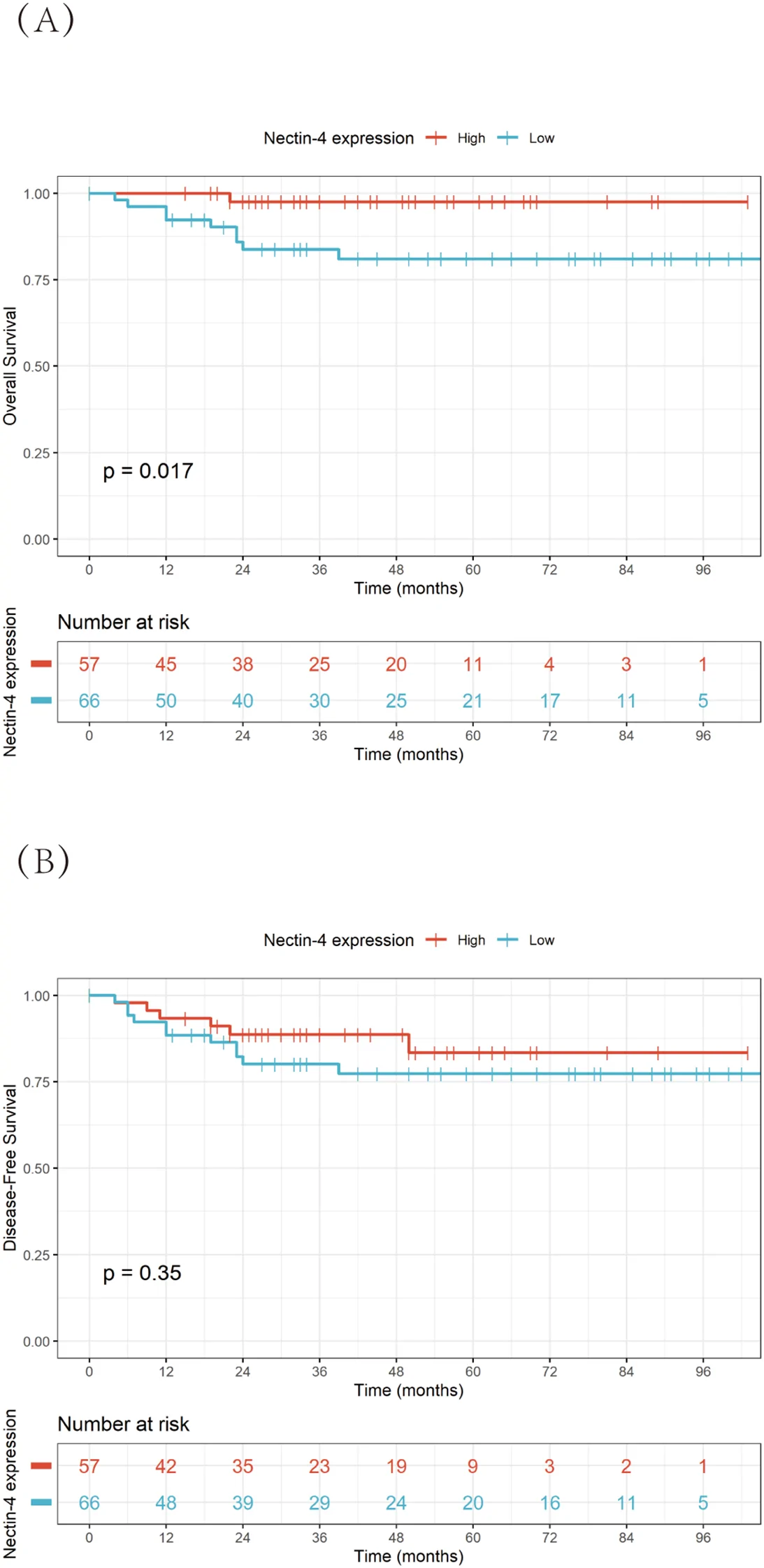
Association of Nectin-4 expression with survival outcomes in PSCC. Kaplan–Meier curves for overall survival **(A)** and disease-free survival **(B)** according to Nectin-4 expression level.

**Table 2:** Univariate and multivariate Cox regression analyses for overall survival in patients with PSCC.

Variable	Univariate analysis			Multivariate analysis		
	HR	95% CI	P-value	HR	95% CI	P-value
Age, ≥61 vs <61 years	0.443	0.114-1.715	0.238			
T stage, T3–4 vs T1–2	3.417	0.881-13.253	0.076			
N stage, N1–3 vs N0	12.631	2.680-59.536	0.001	10.627	2.197-51.392	0.003
Histological grade, G2–3 vs G1	7.160	0.906-56.600	0.062			
H-score	0.951	0.905–1.000	0.048	0.995	0.989–1.001	0.108

CI, confidence interval; HR, hazard ratio; PSCC, penile squamous cell carcinoma.

## 4 Discussion

This study evaluated Nectin-4 expression in primary PSCC and matched lymph node metastases and identified three principal findings. First, Nectin-4 expression was associated with T stage and histological differentiation. Expression was significantly higher in T1 tumors than in T2 tumors and was higher in well-differentiated tumors, suggesting a relationship with retained epithelial differentiation. However, the comparison between G1 and G3 tumors was based on only five G3 cases and should therefore be regarded as exploratory. Second, paired analysis demonstrated significantly lower Nectin-4 expression in lymph node metastases than in the corresponding primary tumors. Third, low Nectin-4 expression was associated with shorter OS in Kaplan–Meier and univariable Cox analyses, but this association was not statistically significant after multivariable analyses. Therefore, the principal contribution of this study is the characterization of primary–metastatic discordance in Nectin-4 expression.

Compared with previous studies of Nectin-4 expression in PSCC, our analysis provides additional information on paired primary and metastatic lesions. Grass et al. and Tekin et al. analyzed relatively small cohorts and focused mainly on primary tumors (14, 15), whereas our study included a larger cohort and allowed a more comprehensive assessment of both primary and metastatic lesions. In addition, although Mink et al. and Tekin et al. included metastatic sites, considerable heterogeneity remained across studies in antibody selection, scoring methods, and positivity thresholds (13, 19). This variability likely contributed to inconsistent estimates of positivity rates and expression levels. Our study used a standardized IHC protocol, whole-section specimens and a uniform H-score-based assessment system, which may have improved analytical consistency. Furthermore, most previous studies did not include paired analyses of primary and metastatic lesions, which limited direct evaluation of how Nectin-4 expression changes during tumor progression and metastasis. By incorporating paired analyses, our study offers more direct evidence that Nectin-4 expression decreases during disease progression.

The association between Nectin-4 expression and tumor differentiation in PSCC is biologically plausible because Nectin-4 participates in epithelial cell-cell adhesion. However, the clinical meaning of Nectin-4 is context dependent. In cutaneous squamous cell carcinoma, experimental silencing of Nectin-4 weakened cell-cell attachment and increased migration-related features while reducing proliferation (20). In esophageal cancer, by contrast, high Nectin-4 expression has been associated with invasion, tumor progression, and poorer prognosis (21). These apparently divergent observations indicate that Nectin-4 cannot be classified simply as either a favorable differentiation marker or a universal driver of aggressiveness across squamous malignancies. In PSCC, our data are most consistent with Nectin-4 serving as a marker of retained epithelial differentiation, but mechanistic studies are required to determine whether its reduction actively contributes to dissemination.

Emerging evidence concerning lineage plasticity in urothelial carcinoma provides a relevant framework for interpreting these findings. In a transcriptomic analysis of urothelial carcinomas with divergent differentiation and variant histology, Luo et al. found that tumors with neuroendocrine, sarcomatoid, and squamous histology exhibited the lowest Nectin-4 expression, whereas micropapillary, plasmacytoid, and nested variants that retained a luminal urothelial transcriptional program generally maintained higher expression levels (22). These findings suggest that the abundance of Nectin-4 and other antibody–drug conjugate targets is partly determined by lineage identity and may decrease as tumors undergo dedifferentiation or phenotypic reprogramming. Although PSCC is biologically distinct from urothelial carcinoma with squamous differentiation, the association between reduced Nectin-4 expression and poorer differentiation in our cohort raises the possibility that similar lineage-state-dependent regulation may operate in PSCC. However, because our study did not assess transcriptional lineage programs, this interpretation remains hypothesis-generating.

Other studies of urothelial carcinoma have demonstrated marked heterogeneity in Nectin-4 expression across histological variants and between primary and metastatic sites (17, 18, 23, 24). Of particular relevance, Klümper et al. evaluated 137 paired primary and metastatic urothelial carcinomas and demonstrated a significant reduction in membranous Nectin-4 expression in metastatic lesions, with 39.4% of metastatic lesions lacking membranous expression. In a separate cohort of patients treated with enfortumab vedotin (EV), absent or weak membranous expression was associated with shorter progression-free survival during treatment (18). These observations support two concepts relevant to our findings: first, Nectin-4 expression may change during metastatic progression; second, target expression in an archival primary tumor may not accurately represent its abundance in current metastatic disease. Nevertheless, these urothelial carcinoma data cannot be directly extrapolated to PSCC, and the relationship between Nectin-4 expression and EV response remains unestablished in penile cancer.

Several non-mutually exclusive mechanisms could explain the lower Nectin-4 expression observed in lymph node metastases. First, metastatic dissemination may select pre-existing tumor subclones with lower Nectin-4 expression, weaker epithelial adhesion, and greater migratory capacity (20). Second, partial epithelial-to-mesenchymal transition or broader lineage plasticity may reduce the expression of epithelial surface proteins without requiring complete conversion to a mesenchymal phenotype (25). Third, immune, stromal, and cytokine signals within the lymph node microenvironment may induce reversible phenotypic remodeling (26). Finally, spatial heterogeneity within primary tumors and differences in tissue sampling may produce apparent primary–metastatic discordance if different tumor subclones are represented in the two specimens (27). Because the present study did not assess EMT markers, transcriptional lineage programs, Nectin-4 copy-number alterations, or clonal relationships, these mechanisms should be considered plausible biological explanations rather than demonstrated causal mechanisms.

The reduction in metastatic Nectin-4 expression creates an important therapeutic tension. Although expression was lower in lymph node metastases, it was not completely lost in most cases, and a subset of metastatic tumors retained intermediate-to-high H-scores. This partial retention provides a rationale for the continued clinical evaluation of Nectin-4-directed antibody–drug conjugates (ADCs) in advanced PSCC, particularly given the limited systemic treatment options available for this disease and the reported clinical activity of EV in an individual patient with metastatic PSCC (28). However, residual immunohistochemical staining alone should not be interpreted as evidence of therapeutic sensitivity. ADC activity may also depend on membranous localization, antigen density, intratumoral homogeneity, receptor internalization, payload sensitivity, drug delivery, and the bystander effect (29). Furthermore, no standardized Nectin-4 IHC assay or response-predictive H-score threshold has been validated in PSCC (13, 15). Therefore, our findings support further biomarker-integrated clinical trials but do not establish Nectin-4 expression as a clinically validated predictor of EV benefit.

At present, routine Nectin-4 IHC testing cannot be recommended for all patients with PSCC. Nevertheless, testing may have value as an exploratory stratification tool in clinical trials and prospective translational studies of Nectin-4-directed treatment. When tissue from a recent metastatic or progressive lesion is already clinically available, assessment of that specimen may more accurately reflect the current therapeutic target status than assessment of the archival primary tumor. Primary-tumor tissue remains a pragmatic alternative when metastatic tissue is unavailable or when additional biopsy would be unsafe or clinically unjustified, but its expression level may overestimate that of metastatic disease. Importantly, our results do not support routinely obtaining a new metastatic biopsy solely for Nectin-4 testing. Prospective studies should evaluate paired primary and metastatic samples using standardized membrane-specific scoring and correlate expression with actual treatment response before Nectin-4 IHC can be incorporated into routine clinical decision-making. The recommendation to prioritize clinically available metastatic tissue is biologically supported by the urothelial carcinoma study showing reduced metastatic membrane expression and poorer EV outcomes in tumors with absent or weak expression, but it remains unvalidated in PSCC.

Low Nectin-4 expression was associated with shorter OS, and high expression was associated with a reduced risk of death in univariable Cox analysis. However, this relationship did not remain statistically significant after adjustment and Nectin-4 expression was not significantly associated with DFS. Reduced expression may therefore reflect adverse pathological characteristics rather than provide independent prognostic information. The wide confidence intervals in the Cox models further suggest limited statistical precision, likely related to the small number of survival events. Accordingly, Nectin-4 should be described as having potential prognostic relevance rather than as an established independent prognostic biomarker.

This study has several limitations. First, its single-center retrospective design introduces potential selection bias and limits generalizability. Second, although the paired design is a major strength, the metastatic cohort comprised only 30 cases, which may limit the precision and reproducibility of the estimated primary–metastatic difference. Third, HPV or p16 status, detailed histological subtype and extranodal extension were not incorporated, although these factors may contribute to biological heterogeneity. Finally, none of the patients in this cohort was evaluated for response to Nectin-4-directed treatment; therefore, the present study is descriptive and prognostic rather than predictive. Multicenter studies incorporating standardized membrane-focused IHC, molecular characterization, and prospective treatment-response correlations are required to clarify the biological and clinical significance of Nectin-4 in PSCC.

## 5 Conclusion

This study shows that Nectin-4 expression in PSCC is associated with clinicopathological features. Nectin-4 expression was higher in lower T-stage and well-differentiated tumors, but was reduced in lymph node metastases. Low Nectin-4 expression was associated with shorter overall survival in unadjusted analyses but was not independently significant after multivariable adjustment. Although Nectin-4 expression decreased in metastatic lesions, it was not completely lost in most cases, indicating that a subset of advanced or metastatic PSCC may retain potentially targetable Nectin-4 expression. Further multicenter validation and mechanistic studies are needed to clarify the clinical utility of Nectin-4 and to define which patients may benefit from Nectin-4-directed therapeutic strategies.

## Data Availability

The original contributions presented in the study are included in the article/**Supplementary Material**. Further inquiries can be directed to the corresponding authors.
