# Supplementary material for: Reduced Nectin-4 expression in lymph node metastases of penile squamous cell carcinoma: a paired primary–metastatic analysis
Source: Front Oncol. 2026 Sep 22;16:1912533. doi: 10.3389/fonc.2026.1912533 (PMC13638354; doi:10.3389/fonc.2026.1912533)
Supplement: Supplementary file 1 [file Supplementaryfile1.docx]

Supplementary Material

# 1 Supplementary Figures and Tables

**Supplementary Table 1.** Characteristics of the 30 paired primary–metastatic cases

|  | Primary tumors, n | Lymph node metastases, n |
| --- | --- | --- |
| Age (years), mean ± SD | 58.27±11.335 | |
| Smoking | 17 | |
| Underlying disease  Hypertension  Diabetes  Coronary artery disease | 6  8  3 | |
| Surgery procedure  Partial penectomy  Total penectomy  Wide excision | 28  1  1 | |
| Regional nodal management  ILND  ILND + PLND | 28  2 | |
| Primary tumor  T1  T2  T3  T4 | 10  15  4  1 | |
| pN stage  N1  N2  N3 | 11  14  5 | |
| Histological grade  G1  G2  G3 | 10  17  3 | |
| Proportion of positive tumor cells  0-25%  25-50%  50-75%  75-100% | 4  2  5  19 | 6  6  5  13 |
| H-score  0-100  100-200  200-300 | 11  13  6 | 17  12  1 |

**Supplementary Table 2.** Numbers of OS and DFS events according to Nectin-4 expression

|  | OS | DFS |
| --- | --- | --- |
| Analysis population, n | 123 | 123 |
| Low Nectin-4, n | 66 | 66 |
| Events in low group, n | 15 | 16 |
| High Nectin-4, n | 57 | 57 |
| Events in high group, n | 7 | 13 |
| Total events, n | 22 | 29 |

## 1.1 Supplementary Figures

**Supplementary Figure 1.** Flow diagram of patient selection. A total of 189 patients with pathologically confirmed penile squamous cell carcinoma who underwent surgery between March 2018 and December 2024 were screened. After excluding 66 patients because primary-tumor FFPE blocks were unavailable (n = 50), neoadjuvant therapy had been administered (n = 7), or specimen preservation or quality was inadequate for analysis (n = 9), 123 patients were included in the primary-tumor cohort. Among 35 patients with pathologically confirmed regional lymph node metastasis, metastatic lymph node FFPE blocks were unavailable in five patients; therefore, 30 patients constituted the paired primary–metastatic cohort. FFPE, formalin-fixed, paraffin-embedded.

**Supplementary Figure 2.** Representative immunohistochemical staining intensities of Nectin-4 in penile squamous cell carcinoma. Representative images showing absent/negative membranous staining (0; A), weak membranous staining (1+; B), moderate membranous staining (2+; C), and strong membranous staining (3+; D).
